# Caregiver's Perspectives on the Healthcare Experiences of Children With Behaviour-Related Disorders

**DOI:** 10.7759/cureus.22084

**Published:** 2022-02-10

**Authors:** Naythrah Thevathasan, Kathryn E Flood, Alison Luke, Sarah A Campbell, Shelley Doucet, Sarah Gander

**Affiliations:** 1 Pediatrics, Dalhousie Medicine New Brunswick, Saint John, CAN; 2 Social Research, Saint John Regional Hospital, Saint John, CAN; 3 Research, University of New Brunswick, Saint John, CAN; 4 Pediatrics Research, Saint John Regional Hospital, Saint John, CAN; 5 Nursing, University of New Brunswick, Saint John, CAN; 6 Pediatrics, Saint John Regional Hospital, Saint John, CAN

**Keywords:** child behaviour and development, relationship building, timeliness, advocacy, social pediatrics

## Abstract

Objective: Social Pediatrics focuses on targeting and mitigating the effects of the social determinants of health on a child’s well-being and development. Negative health outcomes have been seen in children who have faced poverty, food insecurity, inadequate housing, and traumatic events. In particular, children who come from socioeconomically disadvantaged households are more likely to develop behavioural problems. The purpose of this study is to explore the experiences of caregivers for children with a behaviour-related disorder. This includes children affected by attention, academic, and social issues (e.g. attention-deficit hyperactivity disorder, autism spectrum disorder). This study will aim to understand the strengths, barriers, and social limitations to accessing and receiving care for children with behavioural disorders.

Methods: A qualitative descriptive design was used to conduct three focus groups. Of the 64 caregivers contacted, a total of 13 participants agreed to be in the study. All focus groups were analyzed using inductive thematic analysis.

Results: Preliminary findings suggest that caregivers value pediatricians who spend time, communicate, and make a human connection with their patients. Barriers included physician turnover, long wait times for referrals, and a lack of knowledge regarding services and programs available in their area. Three major themes emerged from this study including (1) timeliness to care, (2) advocacy, and (3) relationship building.

Conclusion: Findings suggested that caregivers valued pediatricians who spend time to make a human connection with their patients. Barriers included physician turnover, long wait times for referrals, and a lack of knowledge of available services. Caregivers who were young mothers felt an added layer of judgement when accessing the necessary care for their children. This study is important as it contributes to our knowledge on the role Social Pediatrics can play in the care of children with behaviour-related disorders.

## Introduction

Social Pediatrics is a discipline that focuses on the social determinants of health to mitigate the toxic effects of trauma on a child's well-being and development [1]. Adverse health outcomes have been seen in individuals who have faced poverty, food insecurity, inadequate housing, and traumatic events [1]. Social Pediatrics takes a community-level approach in addressing the needs of children and families in an individual, family, and societal context [1,2]. Children from socioeconomically disadvantaged households are more likely to develop behavioural problems; that is, family socioeconomic status and lone-parent family status are reliable predictors of behavioural problems later in life [3]. Other factors, such as social incompetence, inadequate daily living skills, child health problems, negative life events, emotional issues, behavioural issues, and parental mental health problems within the family, are strong predictors of behavioural disorders in children [4,5].

Diagnoses of behavioural conditions, such as attention-deficit/hyperactivity disorder (ADHD), have become alarmingly widespread among today's children and youth [6]. These behavioural disorders affect academic achievement, social interaction, and the child's quality of life [7]. This is reasonable given that ADHD is characterized by impulsivity, inattention, and hyperactivity [8]. Further, children who have experienced trauma, toxic stress, and challenging social conditions, like poverty and food insecurity, have been shown to mirror symptoms of ADHD or autism spectrum disorder (ASD) [9,10].

New Brunswick has been recognized as a leader in Social Pediatrics and implementing an Integrated Service Delivery framework to help children and youth with complex care needs [1]. In 2017, the Government of New Brunswick launched its family plan to improve timely access to appropriate services and collaboration across care pathways [11]. A significant need for children and youth who face behaviour-related issues includes timely access to diagnoses and care [12]. Despite collaborative efforts, the current model of care poses issues of concern.

Children and families often wait six months to a year for their pediatric appointment, only to be told that they should be receiving care elsewhere (e.g., occupational therapists, speech-language pathologists, social workers, psychologists, psychiatrists). This misalignment between caregivers and providers can result in strained therapeutic relationships and further delays to treatment for the child [13-16]. Treatment for disorders like ADHD is integral to the child's development because, if left untreated, the overall prognosis for these children is poor (e.g., poor academic achievement and low social functioning) [7]. Treatment of ADHD involves behavioural therapy, medication management with stimulants, and digital health interventions [17]. Research has also demonstrated that this population often requires services from other health allied professionals, suggesting a level of complexity [12].

The current study explores caregivers' experiences when seeking treatment for their child with a behaviour-related disorder (i.e., children affected by attention, academic, and social issues [e.g. ADHD, ASD] within the healthcare system). This study aims to understand the strengths and barriers to accessing and receiving care for children with behavioural disorders in New Brunswick, Canada.

## Materials and methods

Methods

A qualitative descriptive design was used to explore the experiences of this population [18]. Ethical approval was obtained from the Horizon Health Network (file number 100075). A total of 64 caregivers responsible for a child who received care from a pediatrician in a medium-sized city in New Brunswick were contacted. Caregivers were contacted after filling out a participate interest form available to them after recruitment criteria were met of seeking behavioural disorder diagnosis. A total of 13 caregivers agreed to participate in one of three focus groups.

Data Collection

Three unique focus groups (2-5 participants/group) were conducted lasting 45-60 minutes in length. Interviews took place via Zoom, a videoconferencing service. Zoom was chosen as an appropriate platform to conduct focus groups because of its ease of use, cost-effectiveness, and security and data storage features. Security was maintained on video platforms by taking steps to provide confidentiality, such as creating a unique password to access the interview, allowing verified emails to enter the group, and locking the virtual meeting room during the focus group. Participants were able to view and hear each other speak. The interview guide highlighted questions around positive and negative experiences when dealing with the healthcare system regarding their child's care, details regarding the due course of referral, diagnosis and treatment, and the effectiveness of communication between the healthcare professionals.

Data Analysis

The data from the focus groups were analyzed independently among members of the research team using the six steps of inductive thematic analysis devised by Braun and Clarke (2006) [18]. Codes from the focus groups were compared between members of the research team to ensure the trustworthiness and internal validity of the results obtained.

## Results

**Figure 1:**
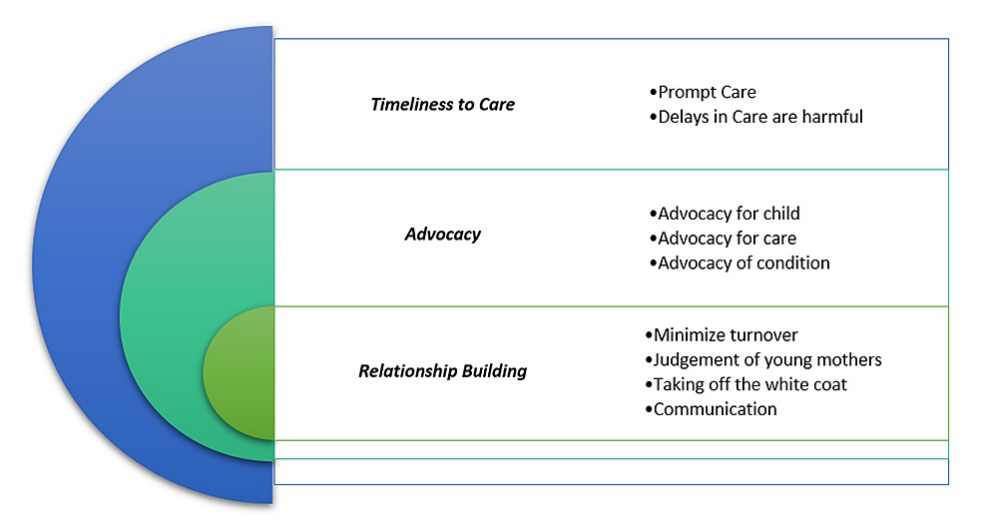
Themes of the perceptions and experience of caregivers with the healthcare system in caring for children with behaviour-related disorders.

Preliminary findings suggest that caregivers value pediatricians who spend time, communicate, and make a human connection with their patients. Barriers included physician turnover, long wait times for referrals, and a lack of knowledge regarding services and programs available in their area. Three major themes emerged from this study including (1) timeliness to care, (2) advocacy, and (3) relationship building (Figure 1).

Timeliness to care

All of the caregivers in the current study valued prompt care. Prompt care was determined by subjective opinion of the caregivers. Many individuals shared that a positive experience in receiving care for their child was how quickly and swiftly care providers responded to their referrals.

"...and similarly getting referrals to Pediatrician and things like that were pretty prompt because of the nature of the issue."

"… but however, when the referral went through when the children were within my care, I felt it was very uh… it was very timely. Um… and, and even more so because I had asked for, you know, both children to see the same Pediatrician, right? So um… I felt the time and that went fairly well. I think it was within, I think, well I should say fairly well but eight weeks and I think for going into that… into a specialty it's good."

However, some caregivers expressed a degree of frustration when there was a lack of timeliness to care. Caregivers felt a significant impact on their child's ability to function and succeed both socially and academically when a delay in care existed.

"In XXX's first school year, she was in French Immersion and she struggled with it a bit and with other kids and what not, but in grade two, we put her into English and her attention span… she doesn't sit still… she doesn't disrupt the class or anything, she just doesn't… she can't focus. She doesn't get any work done so the teachers were trying to come up with different ways to try and help her with wiggly chairs and letting the kids go under the table on mats and different things to learn differently. The uh…. The school actually put us in touch with… told us to get in touch with our doctor and maybe get in touch with a Paediatrician which he did pretty quickly. The only problem was the waiting time which I guess isn't Dr. XXX's fault so it was just a few months before we could get in so it was more towards June before we could get in and we started ADHD medication."

Throughout our focus groups, caregivers consistently expressed the relief they felt when their children got the appropriate care that they needed. Often, timeliness of care means that there is a relief for the child struggling with his/her behavioural needs and relief for the community surrounding and supporting that child. 

Advocacy

Findings of our focus group suggest that caregivers assume a position of advocacy to gain knowledge and access to get their child the care that he/she needs. Part of this need to advocate results from caregivers feeling overwhelmed by the number of referrals provided to obtain holistic care.

"..I think would be one of my negative experiences is I just seem to be referred to this one and then that one says that one and this one says, well no....um...it's not suitable for her..."

Caregivers often felt that part of their role in caring for their children was to advocate for their child's needs and help direct the next steps in care. At times, caregivers felt that care providers were not listening to them in regard to what their child needed leading to personal frustration and fatigue.

"And like, I was pretty, I was a crazy person…. I actually took like five days off of work just to… and I never take time off work… cause I just couldn't do it anymore… like I was like… how is nobody listening… like how can my kid have all of these diagnoses…."

There were times where caregivers also felt that they needed to assume the role of knowing their child's condition better than anyone else. As a result, caregivers felt better equipped to advocate for proper care for their child. This finding seemed to be more applicable for families with children who required complex care (e.g. requiring multiple services from different sectors) in addition to their behaviour-related disorders.

"Um… and, but when it comes to developmental stuff… or complex needs… if you aren't on your game, things don't happen the way it should… it's just the reality of it. If you can't report things and be on top of it and know like…. I remember being at the IWK and memorizing how many lipids my kid was on when he was in NICU because I need to know more than anybody else about my kid."

The literature emphasizes that children and youth with behaviour-related disorders often require services from other health allied professionals, suggesting a level of complexity [12]. Focus group participants echoed similar sentiments that required them to devote time and energy to understanding the various avenues of care that could help their child.

"I've really devoted the last ten years of my life to knowing more than anybody else about everything to do with my child's needs and knowing every single possible way of getting them the services they need, um… so that I'm not depending on anybody else to do that for me. Um… and so knowing what services exist in New Brunswick and how to get referrals to them and who needs to do the referral um… so that when I go to a doctor, I know to ask for that."

Relationship building

Focus group participants continued to place value on clear communication with care providers. Caregivers shared that they valued having care providers who were present consistently in their child's care. Excessive turnover in staff was hard for caregivers and their children to develop strong therapeutic alliances.

"I think like the only real negative and I live in a small town so doctors… like main doctors, you know family doctors, come and go very quickly so once your family doctor gets to know your child, they seem to be gone and we are on to the next one so…."

Caregivers valued care providers who took the time to come down to their child's level by taking off the "white coat," allowing both families and patients to feel more comfortable. Participants felt that care providers created distance and barriers in terms of the therapeutic relationship without this approach.

"So um… I've explained that to the, the the physician a couple of times with hopes that it would change his approach and not to be so professional… right? Like take the white coat off."

Effective communication is the cornerstone of a positive therapeutic relationship. Caregivers felt that care providers who could communicate not only to them but also to their child bolstered a sense of trust and belonging.

"...throughout [the pediatrician] is great with communicating with them and communicating XXX's needs and then…. Like she talks to the kids. Like we go in there but it's like, "okay, how are you" but wait, I don't want to talk to you, I want to talk to your kid and ask how they're doing and where they're at and what they want to see happen…"

"She is very easy to talk to and down to earth. She talks with you and with the kids. She doesn't talk at you so… anything we need to discuss has been pretty open and easy to have a conversation with her."

An area of need identified among participants was judgement-free relationship building. In particular, young mothers of lone-parent households often felt that their concerns for their child were written off as individual worry. As a result, these participants felt less seen and heard. It was revealed that these feelings of judgement result in fear of accessing the healthcare system and subsequently result in poor loss to follow-up.

“you’ve got parents out there visiting you [the doctor] because they want help, does it matter what their age is? No it shouldn’t, they’re there trying to do the best for their kids, don’t judge them.” 

“[at the hospital] They treated her as a young mom calling her like oh this baby is weightless [inferring malnourished], like, you’re, you’re wrong, like, label after label after label […] so I kind of lost my crap and told them to adjust their scales and walked out and never returned."

## Discussion

The themes identified in our study highlight how caregivers valued timeliness to care, relationship building, and advocacy. However, our themes center around personal experiences at the micro-level of care versus systemic barriers or limitations. Other studies suggest that social disparities impact a child's ability to access care and be assessed for a behaviour-related disorder (for example, being a visible minority, male children, or living in poverty) [19]. Poverty was shown to be the most pervasive barrier to children accessing behavioural interventions and treatment [19]. The findings of our study failed to capture the social determinants of health and systemic disparities that exist for children and their families. One sub-theme that did emerge in our research was the judgement of caregivers who were young mothers of lone-parent homes by care providers. Evidence indicates that lone mother-led families are severely disadvantaged by social and financial inequity [20]. However, this is an inference we did not acknowledge in the structure of our study design.

An extensive study done with caregivers in Europe with children with ADHD highlighted that a third of caregivers found it challenging to obtain a diagnosis of ADHD [21]. This particular study noted that the time to onset of symptoms to first doctor's visit was two to three times longer than the first initial visit with the doctor to the point of diagnosis [21]. This subtle finding is striking as it also highlights the theme of timeliness to care, which was found in our study. Part of the disparity regarding timely care can be attributed to long wait times, lack of specialists, and co-morbid conditions that mask initial behavioural symptoms [22]. The importance of timely care for children with detrimental behaviour-related disorders is particularly critical regarding exacerbating poor educational and psychological outcomes [22]. When children have delayed diagnoses, it has manifested in higher depression and low self-esteem [23]. In our study, most caregivers were appreciative regarding the timely access to care that they had received. However, those that expressed frustration did highlight issues with poor educational and social outcomes for their child.

A central theme in this study was the emphasis on caregiver advocacy for their child and their condition. Advocacy is seen as an emerging parental task that is not highlighted much in the literature despite its importance. A study done with caregivers of children with ASD showed that parental stress related to advocacy was rated more than stress associated with activities related to daily living for their child (e.g. bathing, toileting, dressing, etc.) [23]. It is interesting to contrast these findings to findings in our study as caregivers emphasized the importance and value of knowing and advocating for their child but failed to comment on the subsequent stress this role may play personally and on the family unit.

Caregiver-related stress in caring for children with emotional and behavioural difficulties has been well documented [24]. It has been shown that the complexity of a child's behavioural needs is likely proportional to the caregiver stress felt [25]. Therefore, it has been proposed that in the same way we provide support and intervention for the child affected by behaviour-related disorders, we must do the same for caregivers involved [25]. Caregivers take on an enormous burden of responsibility when coordinating the care for their child, which sometimes results in life-altering decisions (e.g. quitting a job to stay home with a child) [26]. As a result, it is essential to recognize the avenues to create changes at the macro-level of care to better support caregivers. One avenue that has been explored in this regard is patient navigation. Patient navigation has been shown to connect patients and caregivers to resources; streamline care; facilitate transitions in care; and provide education, advocacy, and support [27-30].

At a micro-level, our study continues to reinforce the value of the caregiver and care provider relationships. Strains between receiving adequate care from care providers in the context of behaviour-related disorders are still an issue [19]. A study on caregiver perspective on ADHD diagnosis and care in Europe noted that half of the caregivers were dissatisfied with their children's care. It has been hypothesized that this may be due to a lack of care provider knowledge and coordination among members of the care team offering different services [20]. Our study highlighted the need for good communication and having care providers come down to the child's level. Good physician communication may increase patient satisfaction with care, increase physician and patient understanding, and improve autonomy and ownership of treatment plans [30]. Children with complex care needs require services that are both varied and integrated across different allied health sectors [12]. Social Pediatrics plays a role in bridging care that is intersectional, integrated, and focuses on both the child and community [30]. This is known as the RICHER model of care, a way in which children with behaviour-related disorders can be served [30]. An example of such care can include patient navigation to improve access and coordinate care. This insight may propose that good relationship-building between caregivers and care providers could mediate the limitations that exist with the healthcare system and should be investigated further.

Limitations

While the findings of this study provide insight into the issues that exist for caregivers in seeking care for their children with behaviour-related disorders, it does also have its own set of limitations. This study highlights the experiences of caregivers within the Saint John catchment during a limited sampling time frame. Of the 64 caregivers who were contacted, only 13 were able to participate in the study. Responses were largely dependent on participant recall and recollection. It is unknown if the participants who participated did so solely because their experiences were positive.

Systemic and social factors continue to be highlighted in the literature as barriers to care for children with behaviour-related disorders [16]. In our study, we did not collect nor highlight the specific social determinants of health that could have impacted a caregiver's experience in accessing care. Therefore, it is hard to infer and conclude social and systemic barriers that could have played a role in the experiences of these caregivers.

In addition, this study was conducted during the peak of the COVID-19 pandemic. As a result, participants who did participate were also those who had access to technology to do so. We had offered participants the ability to join at a third-party site if they showed interest in participating but did not have the means to do so. Another limitation of the study was that all of the focus groups were conducted virtually online. The convenience of a virtual platform helped us navigate the limitations of social distancing during the pandemic, limited the richness of communication and community during this study, and altered our ability to conduct the focus groups in a controlled environment with minimal distraction.

## Conclusions

Caregivers play an integral role in understanding their child's needs and communicating those concerns to their care team. This study has highlighted the importance of timeliness to care, advocacy, positive relationship building, and the value of "taking off the white coat" for children with behaviour-related disorders. Early intervention has a key role in the prognosis of a child's development and social functioning at home and in school. Caregivers felt that delays in care may be harmful to the child's overall well-being.

Children with behaviour-related disorders are a complex patient population who requires streamlined and integrated care. This study contributes to our knowledge on the role Social Pediatrics can play in caring for children with complex care needs. Social Pediatrics utilizes and promotes a holistic lens that employs an equitable and interdisciplinary approach that is so needed in children with behaviour-related disorders. 

## Appendices

Focus group discussion guide

Technology Review

Due to the nature of the focus groups being conducted on Zoom, the investigator must ensure that issues of accessibility are addressed. The following questions should be addressed and dealt with accordingly before conducting the focus group to ensure accessibility standards are met.

·       Do you have access to reliable internet?

·       Do you have a device (e.g. tablet, smartphone, computer) that you have access to?

·       Does your device have the facilities to access Zoom?

·       Does your device have a camera option?

·       Do you have access to a quiet space to participate in the focus group?

Consent Process (This Will Be Done Over the Phone During the Confirmation of Participation)

Consent forms for focus group participants are completed in advance by all those seeking to participate.  Below is a summary of the information in the consent form that focus group organizers and facilitators should use to make sure participants understand the information in the consent form.

Thank you for agreeing to participate.  We are very interested to hear your valuable opinion on your family’s experience after your child received a behaviour-related referral. All of the feedback required from the focus groups will be used to finalize the triage tool and pilot phase of the project. Any changes that are made from the current protocol will be subject to amendments through Horizon Health Network’s Research Ethics Board.

The purpose of this study is to learn how experiences after a referral regarding either behaviour, attention, social, or academic issues may vary for different families and different stages of a child’s care. This is so we can work to improve the treatment of children receiving these referrals in a manner that is suitable to all families.

The information you give us is completely confidential, and we will not associate your name with anything you say in the focus group.

We would like to audio record the focus groups so that we can make sure to capture the thoughts, opinions, and ideas we hear from the group.  No names will be attached to the focus groups and the audio tapes will be destroyed as soon as they are transcribed.

You may refuse to answer any question or withdraw from the study at any time.

We understand how important it is that this information is kept private and confidential.  We will ask participants to respect each other’s confidentiality.

If you have any questions now or after you have completed the questionnaire, you can always contact a study team member like me, or you can contact one of the Principal Investigators their contact information is on the consent form.

Introduction:

1.     Welcome

Introduce yourself and the note taker and send the Sign-In Sheet with a few quick demographic questions (age, gender etc.) around to the group while you are introducing the focus group.

Review the following:

·       Who we are and what we’re trying to do

·       What will be done with this information

·       Why we asked you to participate

Do a small ice breaker with the group to help with introductions.

2.     Explanation of the process

Ask the group if anyone has participated in a focus group before.  Explain that focus groups are being used more and more often in health and human services research.         

About focus groups

·       We learn from you (positive and negative)

·       Not trying to achieve consensus, we’re gathering information

·       In this project, we are doing both questionnaires and focus group discussions. The reason for using both of these tools is that we can get more in-depth information from a smaller group of people in focus groups.  This allows us to understand the context behind the answers given in the written survey and helps us explore topics in more detail than we can do in a written survey.           

Logistics

·       Focus group will last about one hour

·       If you need to leave to go to the bathroom you can private message Kate or Naythrah to let them know

·       If there are technical issues, reach out to Kate via private chat (e.g. have them do a sample message just so that they know how to use the feature. This message can include their name, age, gender and any demographic questions we need them to answer.)

·       If you are in an area where you feel like there is or will be a lot of background noise, please mute your mics until it is your time to talk. If noise is not an issue, feel free to keep you mics unmuted to encourage free flowing conversation

3.     Ground Rules

Ask the group to suggest some ground rules.  After they brainstorm some, make sure the following are on the list.

·       Everyone should participate.

·       Information provided in the focus group must be kept confidential

·       Stay with the group and please don’t have side conversations

·       Turn off cell phones if possible

·       Have fun

4.     Turn on Tape Recorder

5.     Ask the group if there are any questions before we get started and address those questions.

6.     Introductions

·       Go around table:  job here, where you were born

Discussion begins, make sure to give people time to think before answering the questions and don’t move too quickly.  Use the probes to make sure that all issues are addressed but move on when you feel you are starting to hear repetitive information.

Questions:

1.     Let’s start the discussion by talking about some good experiences you have had using the health care system in New Brunswick.

2.     What are some experiences you have had that are not so positive?

3.     Have your experiences with your children been positive or negative? Why or why not?

4.     What do you think your child would say about their experience?

5.     Since receiving the referral for your child how long have you had to wait for consultation? Has this been reasonable? What about after diagnosis? Did treatment start immediately? What steps were taken for your child to receive care?

6.     Did you find information was communicated effectively among professionals in your child’s life? (e.g., were you updating the pediatrician on what the family physician said? Or was this done without your help?)

Exit Questions

*Does anybody else have anything to add?

*Have I missed any key issues you would like discuss?

Probes for Discussion:

·       Experiences in the system

o   Positive

o   Negative

o   Is there anything they feel played a role in this?

·       Efficacy of services received

·       The child’s experience

o   What would the child say?

o   Did the child notice?

o   Depending on the stage of treatment how is the child today?

·       Wait times

o   From referral to treatment

o   Among professionals

·       Cohesiveness of system

o   This includes: doctors, nurses, psychologists, teachers, social workers etc.

o   Who is included?

o   Who needs to be included?

o   Was this a problem?

That concludes our focus group.  Thank you so much for coming and sharing your thoughts and opinions with us.  We have a short evaluation form that we would like you to fill out if you have time.  If you have additional information that you did not get to say in the focus group, please feel free to write it on this evaluation form.
